# Supplementary material for: AAV-mediated editing of PMP22 rescues Charcot-Marie-Tooth disease type 1A features in patient-derived iPS Schwann cells
Source: Commun Med (Lond). 2023 Nov 28;3:170. doi: 10.1038/s43856-023-00400-y (PMC10684506; doi:10.1038/s43856-023-00400-y)
Supplement: Supplementary file 1 — Supplementary Information [file 43856_2023_400_MOESM1_ESM.pdf]

AAV-mediated editing of PMP22 rescues Charcot-Marie-Tooth disease type 1A features in patient-derived iPS Schwann cells

Yuki Yoshioka[1,#], Juliana Bosso Taniguchi[1,#], Hidenori Homma[1,#], Takuya Tamura[1], Kyota Fujita[1], Maiko Inotsume[1], Kazuhiko Tagawa[1], Kazuharu Misawa[2,3], Naomichi Matsumoto[2], Masanori Nakagawa[4], Haruhisa Inoue[5,6], Hikari Tanaka[1,$] and Hitoshi Okazawa[1,$]

1: Department of Neuropathology, Medical Research Institute, Tokyo Medical and Dental University, 1-5-45, Yushima, Bunkyo-ku, Tokyo 113-8510, Japan.

2: Department of Human Genetics, Yokohama City University Graduate School of Medicine, Yokohama, Kanagawa 236-0004, Japan

3: RIKEN Center for Advanced Intelligence Project, 1-4-1 Nihonbashi, Chuo-ku, Tokyo 103-0027, Japan.

4: Department of Neurology, Kyoto Prefectural University of Medicine, Kyoto 606-8507, Japan

5: Center for iPS Cell Research and Application (CiRA), Kyoto University, Kyoto, 606-8507, Japan.

6: Drug-discovery cellular basis development team, RIKEN BioResource Center, Kyoto 606-8507, Japan

#: These authors contributed equally

$: Corresponding author (okazawa-tky@umin.ac.jp)

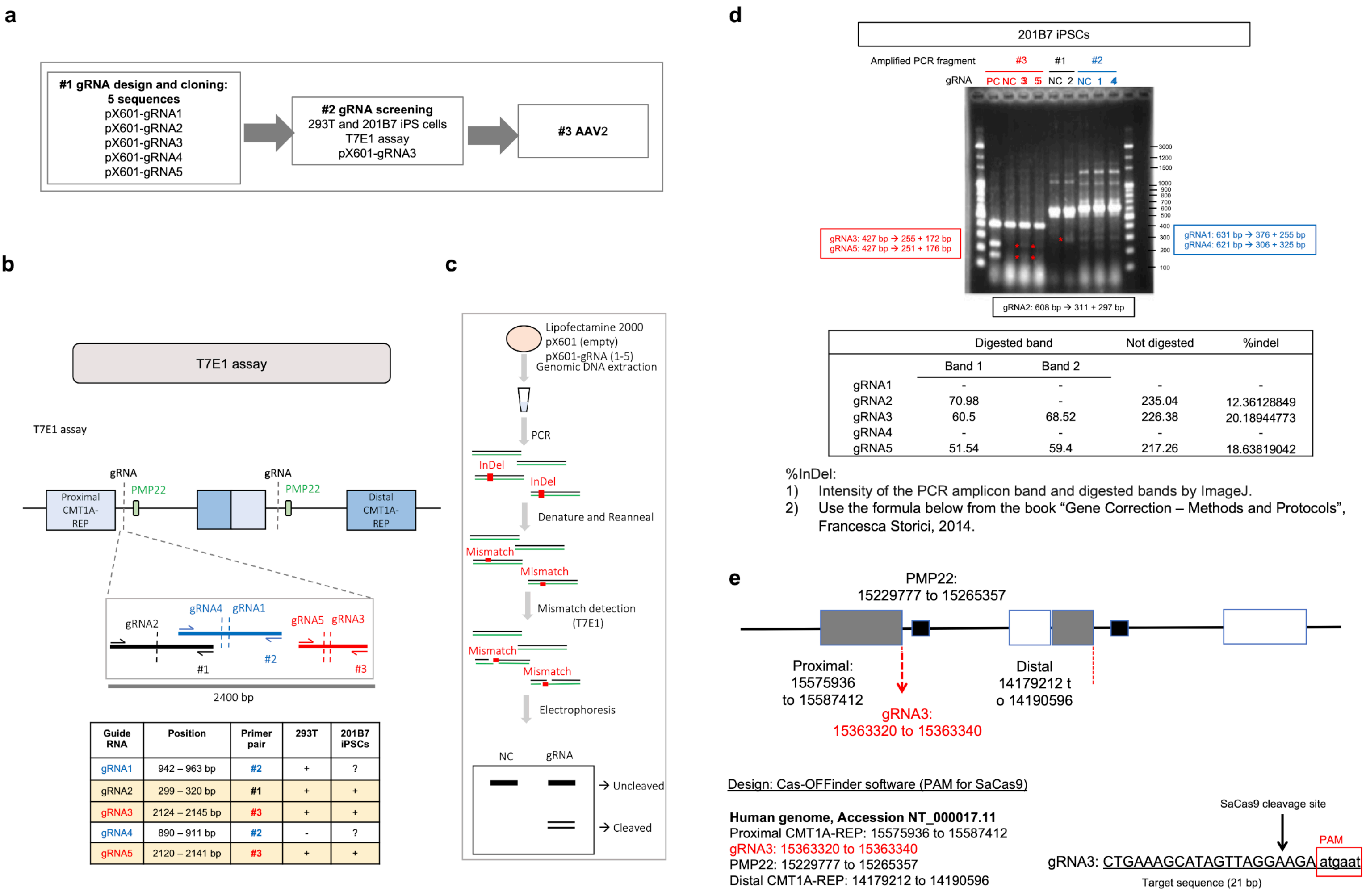

Supplementary Figure 1

Selection of guide RNA sequence for genome editing of duplicate PMP22 gene

a) Schematic diagram of design and screening for guide RNA. Five guide RNA sequences were designed and cloned into a pX601 vector. Plasmids containing guide RNAs were transfected into 293T cells and normal iPSCs for genome editing. A T7Endonuclease1 assay was performed to determine the genome editing efficiency of CRISPR-SaCas9. Guide RNA 3 exhibited the highest cutting efficiency among the five candidates and was selected to be cloned into AAV2.

b) T7Endonuclease1 assay to determine relative gene editing efficiency. Guide RNA sequences were designed in a 2400-bp region flanking proximal CMT1A-REP. Three primer pairs were necessary to perform PCR of the five guide RNAs for the T7E1 assay. In 293T cells, all sequences exhibited detectable activity except for gRNA4. In normal iPSCs, cleavage activity was noticeable in gRNA 2, gRNA 3, and gRNA 5.

c) Schematic representation of T7E1 assay. Plasmids containing guide RNA sequences and plasmids without guide RNA were transfected into cells with Lipofectamine 2000 reagent. After 48 h of incubation, genomic DNA was extracted and PCR with the above primers was performed. PCR products were denatured and reannealed, and DNA from edited cells was reannealed with DNA from non-edited cells to generate a heteroduplex. The reannealed heteroduplex was cleaved by T7E1 endonuclease that detects mismatch of heteroduplex and cleaves double strand DNA at the mismatch region, while homoduplexes remained intact. The resulting fragments were of diff erent lengths and could be differentiated in an agarose gel.

d) Gel images of PCR products from normal iPSCs. Gel band sizes were expected as follows: sgRNA1: 376 and 255 bp, sgRNA2: 311 and 297 bp, sgRNA3: 255 and 172 bp, sgRNA4: 306 and 325 bp, sgRNA5: 251 and 176 bp. The genome editing efficiency of CRISPR-SaCas9 was assessed according to the InDel (Insertion/Deletion Polymophism) mutation rate measured by the intensity of PCR bands. Intensity values are summarized in the table. Cutting was not detectable for gRNA1 or gRNA4. (E) Scheme showing the position of guide RNA sequences within the CMT1A genome. The sequences were drawn in a region outside the CMT1A-REP. PAM sequences were generated with Cas-OFFinder software. In the example of gRNA1 with 21bp, black arrow points indicate SaCas9 cleavage sites and the red box indicates the PAM sequence.

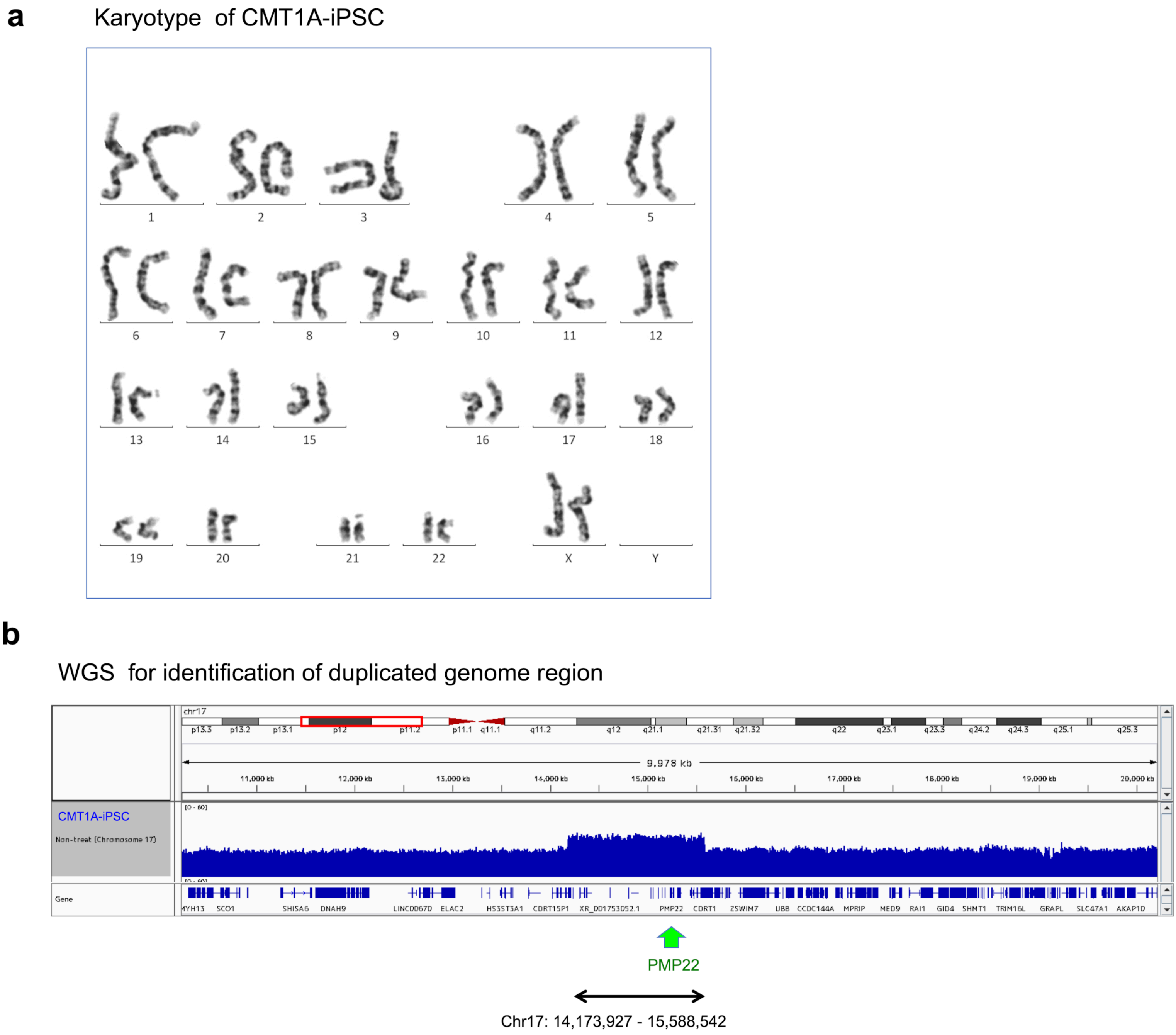

Supplementary Figure 2
Evaluation of CMT1A-iPSC integrity
a) Karyotype analysis of CMT1A-iPSCs (CiRA00139) by G-banding.
b) Whole genome analysis to identify duplicated region. Number of reads is mapped to 17p11.2 genome region.

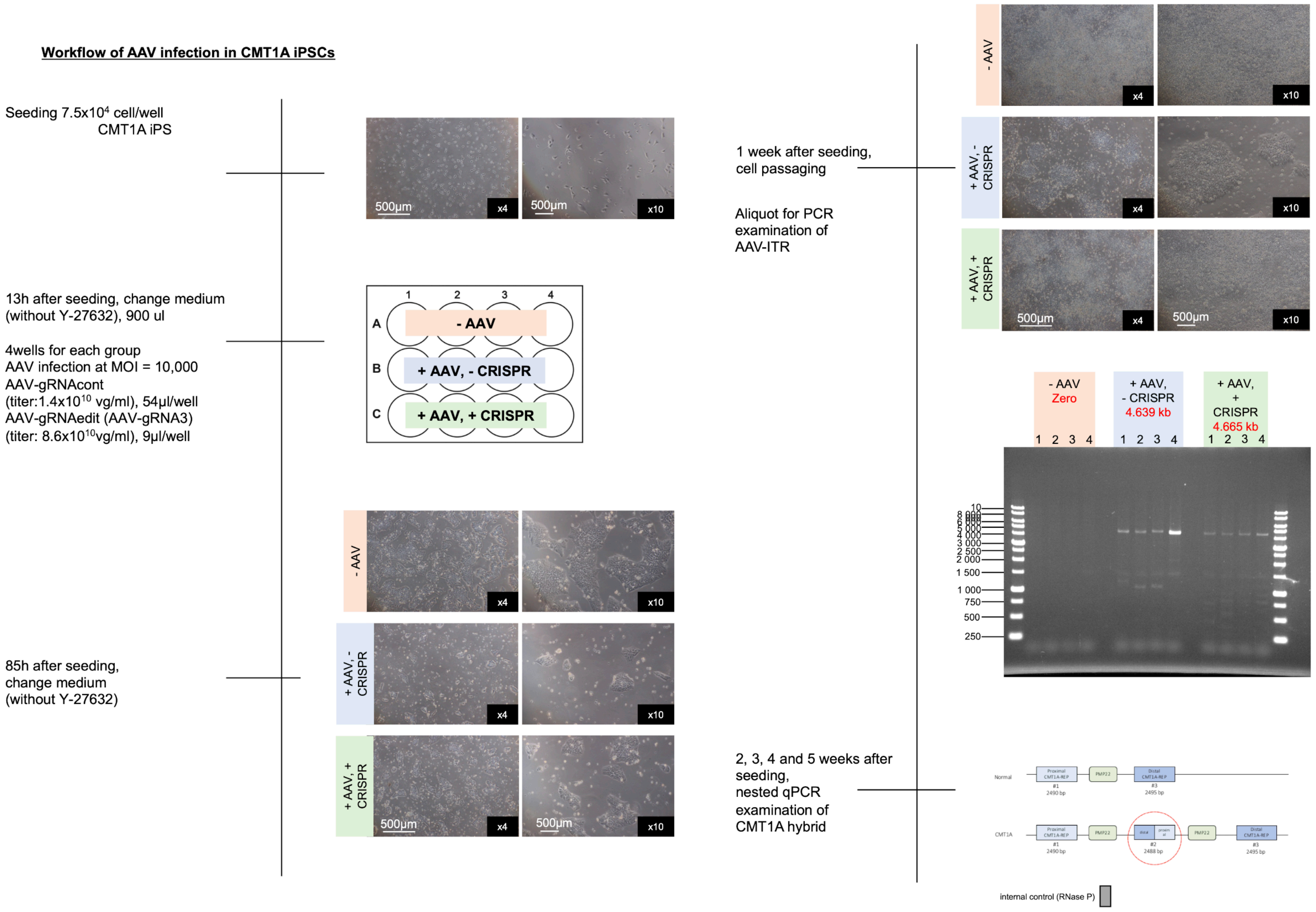

Supplementary Figure 3
Workflow of AAV infection in CMT1A iPSCs

CMT1A patient-derived iPSCs cultured in a feeder-free system were seeded in a Matrigel GFR-coated 12=well plate. After 13 hour, cells were separated into three groups: no AAV infection, infection with AAV without guide RNA, and infection with AAV carrying the guide RNA sequence 3 (MOI =10,000). After 72 h, cells were changed to fresh medium and after 1 week, cells developed confluency necessary for the first passage. On 7th day after AAV infection, virus infection of CMT1A iPSCs was confirmed by PCR amplification of the AAV-ITR region, and samples were prepared for Nested qPCR of the CMT1A hybrid region. RNase P was used as the internal control for Nested qPCR

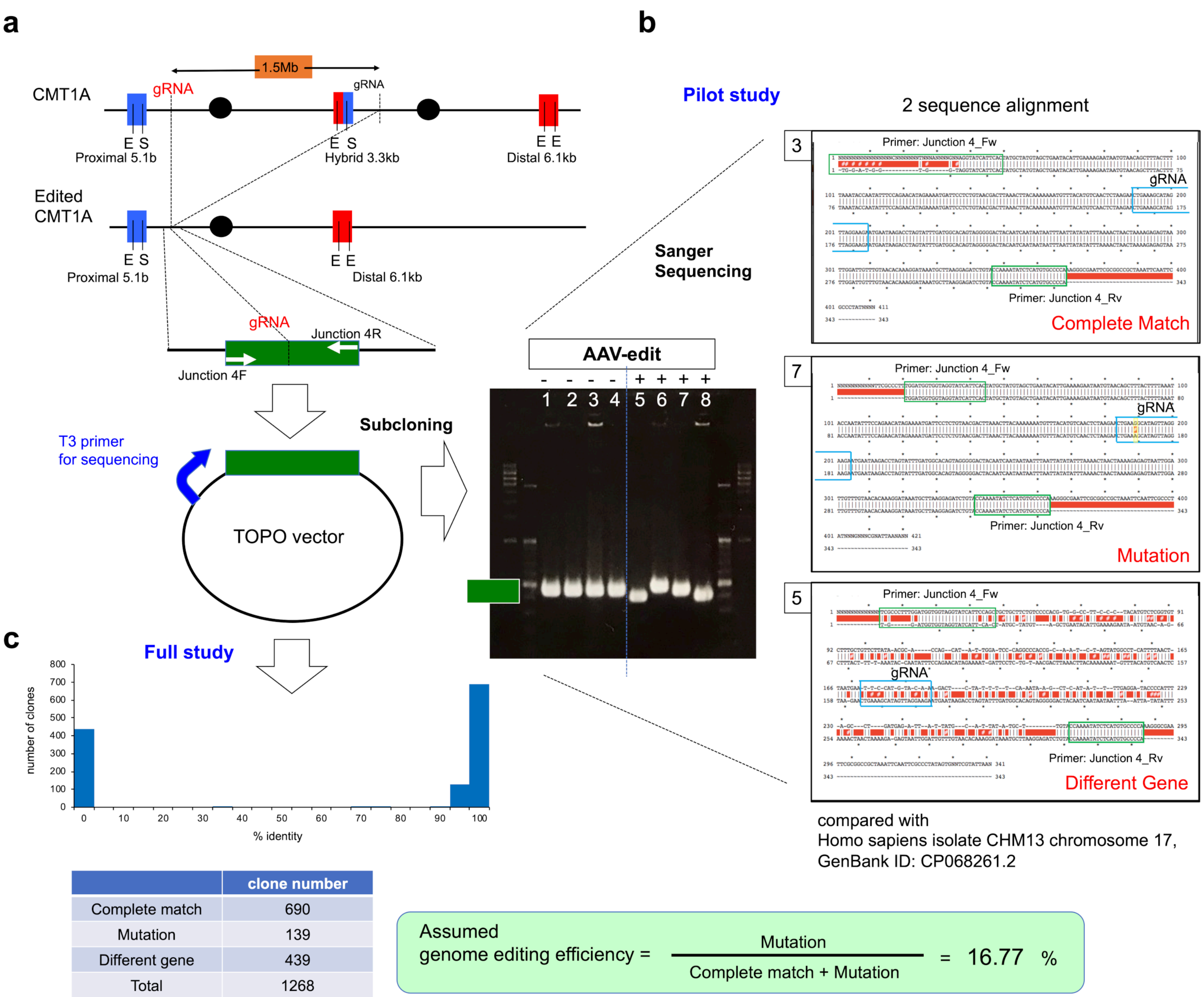

	clone number
Complete match	690
Mutation	139
Different gene	439
Total	1268

$$\text{Assumed genome editing efficiency} = \frac{\text{Mutation}}{\text{Complete match + Mutation}} = 16.77\ \%$$

Supplementary Figure 4

Evaluation of genome editing efficiency by TIDE

a) Method of TIDE, TOPO subcloning and Sanger sequencing. The region around the target sequence of gRNA was PCR-amplified, subcloned into TOPO plasmid and sequenced by Sanger method.

b) Results of pilot study with four plasmids from non-infected iPSCs (without infection) and four plasmids from AAV-gRNAedit-infected iPSCs. Representative cases of TOPO plasmid sequences are shown in right panels. Plasmids 2 and 1 include non-edited genome sequence around the target of gRNA (upper panel). Plasmid 5 contained a completely different sequence that is mapped to a clone RP11-203E6 on chromosome 5 (ID: AC116310.2), indicating that this clone is derived from PCR primer hybridization with non-target genome sequences. Plasmid 7 included a point mutation at gRNA target sequence, which is judged as a result of genome editing.

c) Summary of the full study of 1,268 plasmids. The efficiency of genome editing was calculated to be 16.77 %.

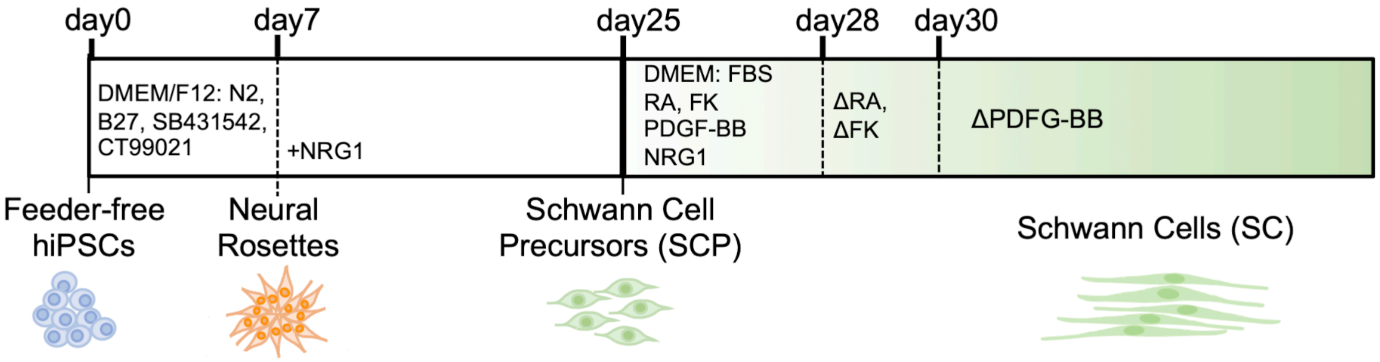

SCP differentiation Medium

1:1
DMEM/F12
Neurobasal

1x N2
1x B27
0.005% BSA
2mM GlutaMAX
0.11mM β-Mercaptoethanol

3μM CHIR
20μM SB

50 ng/mL NRG1

SC differentiation Medium

DMEM low glucose
1% FBS

200 ng/mL NRG1
4μM FK
100nM RA
10 ng/mL PDGF-BB

Supplementary Figure 5
Differentiation protocol from hiPSCs to Schwann cells
Feeder-free hiPSCs cells were initiated in culture in medium containing SB431542 and CT99021 to differentiate into Neural Rosettes. Addition of NRG1 further induced Schwann Cell Precursors. Subsequently, culture medium was replaced with SC differentiation medium to differentiate SCPs into Schwann cells.

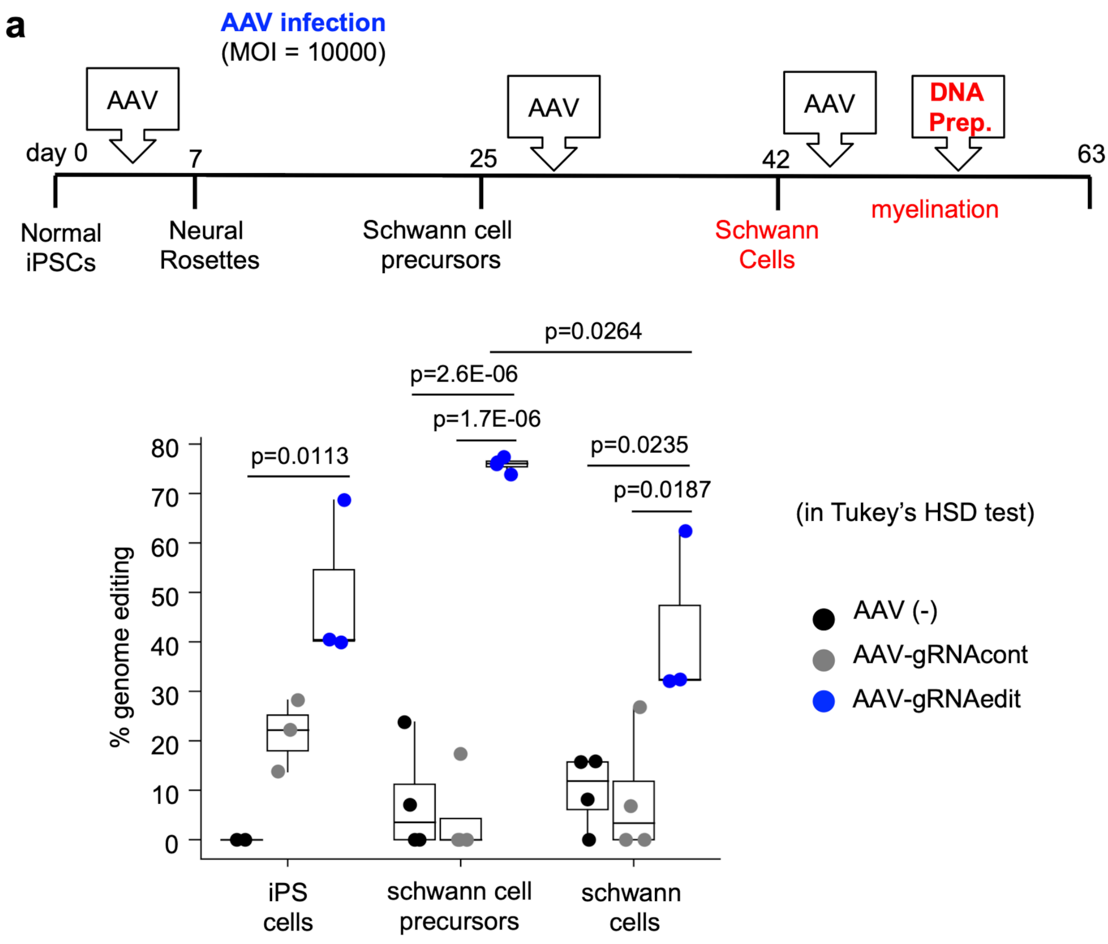

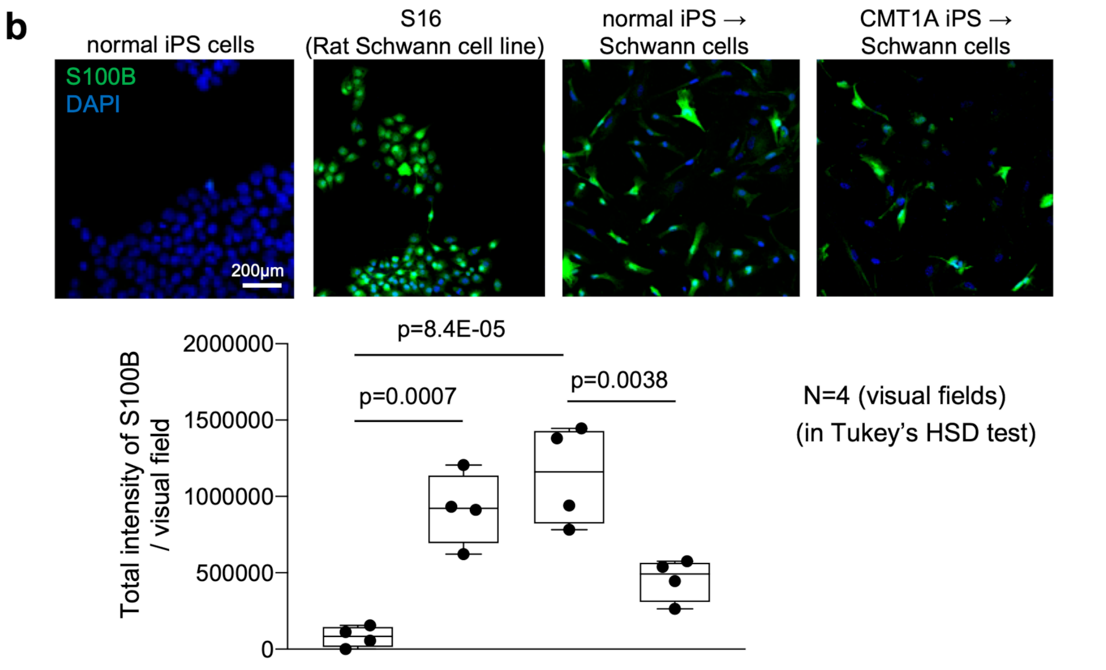

Supplementary Figure 6

Genome editing efficiencies by infection at different time points and Schwann cell differentiation stages in CMT1A-iPSC-derived iPSCs

a) Upper panel shows the protocol. AAV genome-editing vector or AAV control vector was infected at the stages of iPSC, Schwann cell precursors, and mature Schwann cells. Percent genome editing efficiency was evaluated as described in Figure 2. Number of samples (AAV-, AAV-gRNAcont, AAV-gRNAedit) were (2, 3, 3) at iPSCs, (4, 4, 4) at SCPs and (4, 4, 3) at Schwann cells. P-values in Tukey's HSD test are shown.

b) Immunostaining of S100B revealed impaired differentiation of CMT1A-iPSC-derived iPSCs. The lower graph shows quantitative analysis of S100B signal intensity/field. N=4 each. P-values in Tukey's HSD test are shown.

The box plot shows median, 25–75th percentile, and whiskers representing data outside the 25–75th percentile range.

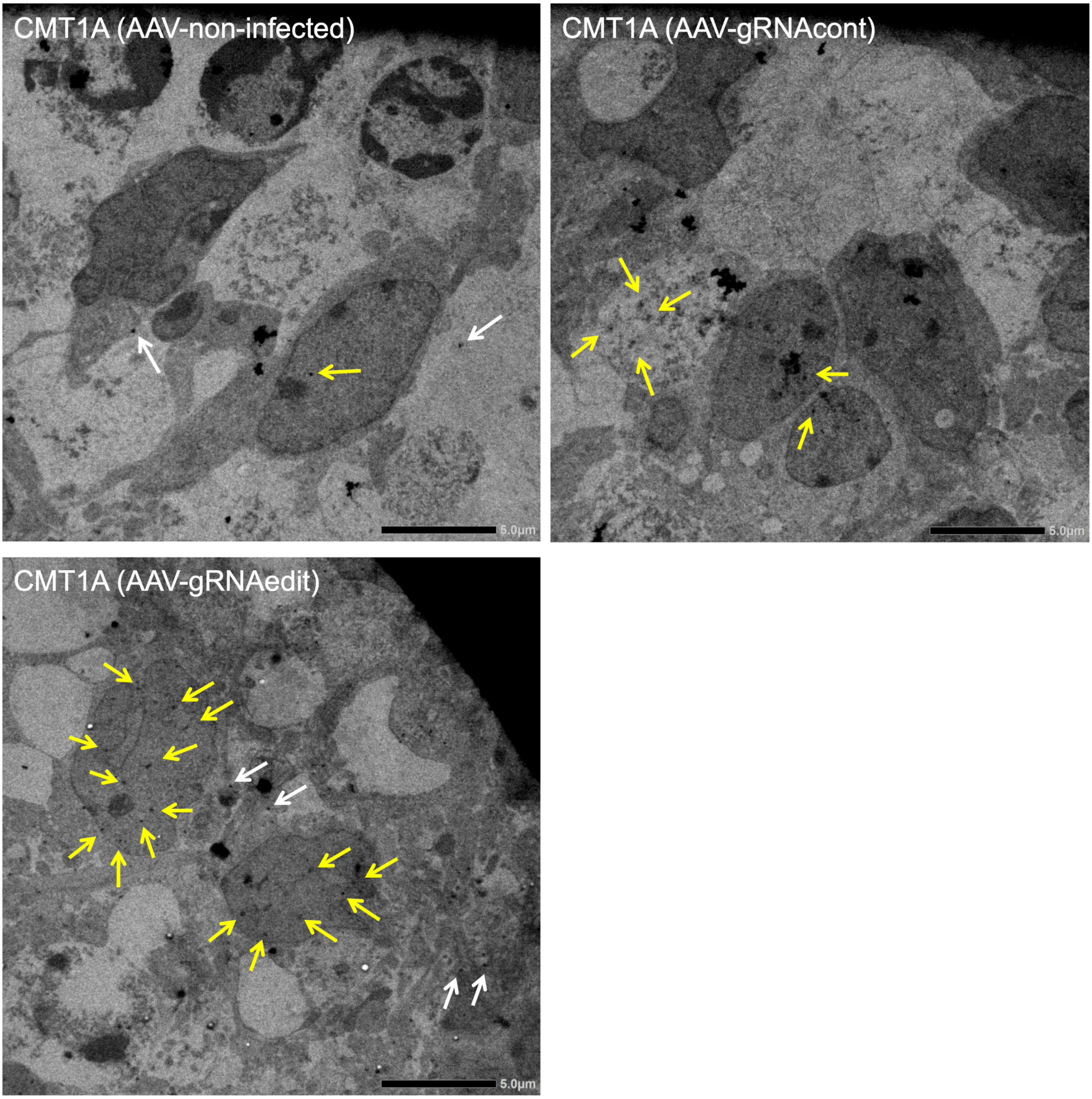

Supplementary Figure 7

Confirmation of AAV genome-editing vector infection by immunoelectron microscopy

Immunoelectron microscopy with anti-Crispr-Cas9 antibody revealed nuclear specific stains (yellow arrows), i.e. gold particles in the nucleus, in CMT1A iPSC-derived Schwann cells, while it detected only a few non-specific stains in the cytoplasm (white arrows) in non-infected CMT1A Schwann cells.

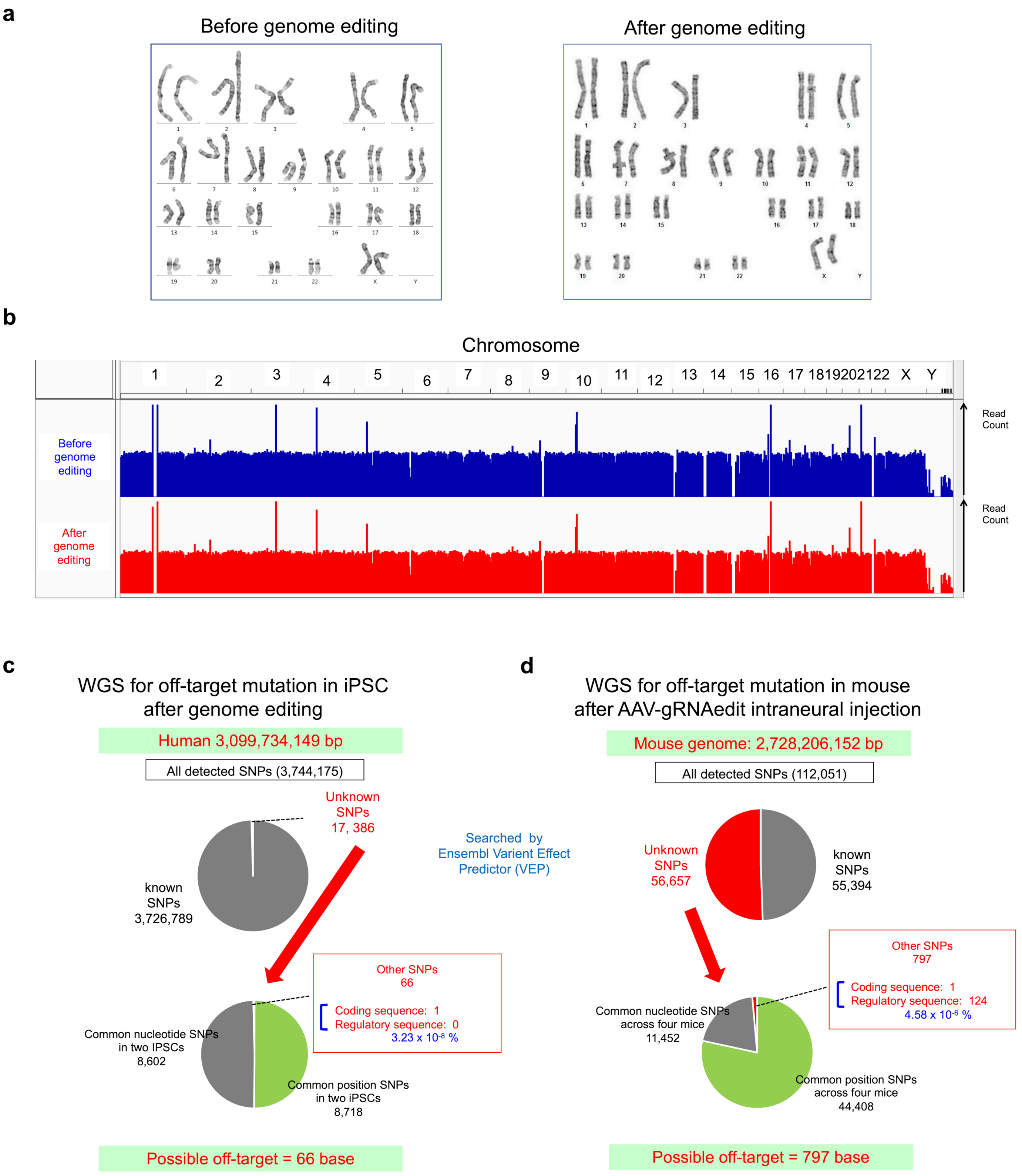

Supplementary Figure 8

Effect of genome editing on chromosome and genome sequence

a) Karyotype analysis of CMT1A-iPSCs after genome editing by G-banding.

b) Genome structure analysis by whole genome sequencing of genome DNA prepared from 1 x 10^6 cells. Genome structures were not changed at the chromosome and CNV levels after genome editing.

c) Whole genome sequencing to identify off-target effects on CMT1A-iPSCs after genome editing by infection of AAV-gRNAedit.

d) Whole genome sequencing to identify off-target effects on peripheral nerve and surrounding tissues after genome editing by intra-neural injection of AAV-gRNAedit.

In regards of "unknown SNPs", "common nucleotide SNPs" mean the SNPs whose position is known but their changed nucleotide is not recorded in SNP database, while "common position SNPs" mean the SNPs whose position is not recorded in SNP database but the changed nucleotide is same between before and after genome editing. Final percentages of possible off-target effects on coding and regulatory regions were 3.23 x 10^{-8} % in human iPSC and 4.58 x 10^{-6} % in mouse tissues.

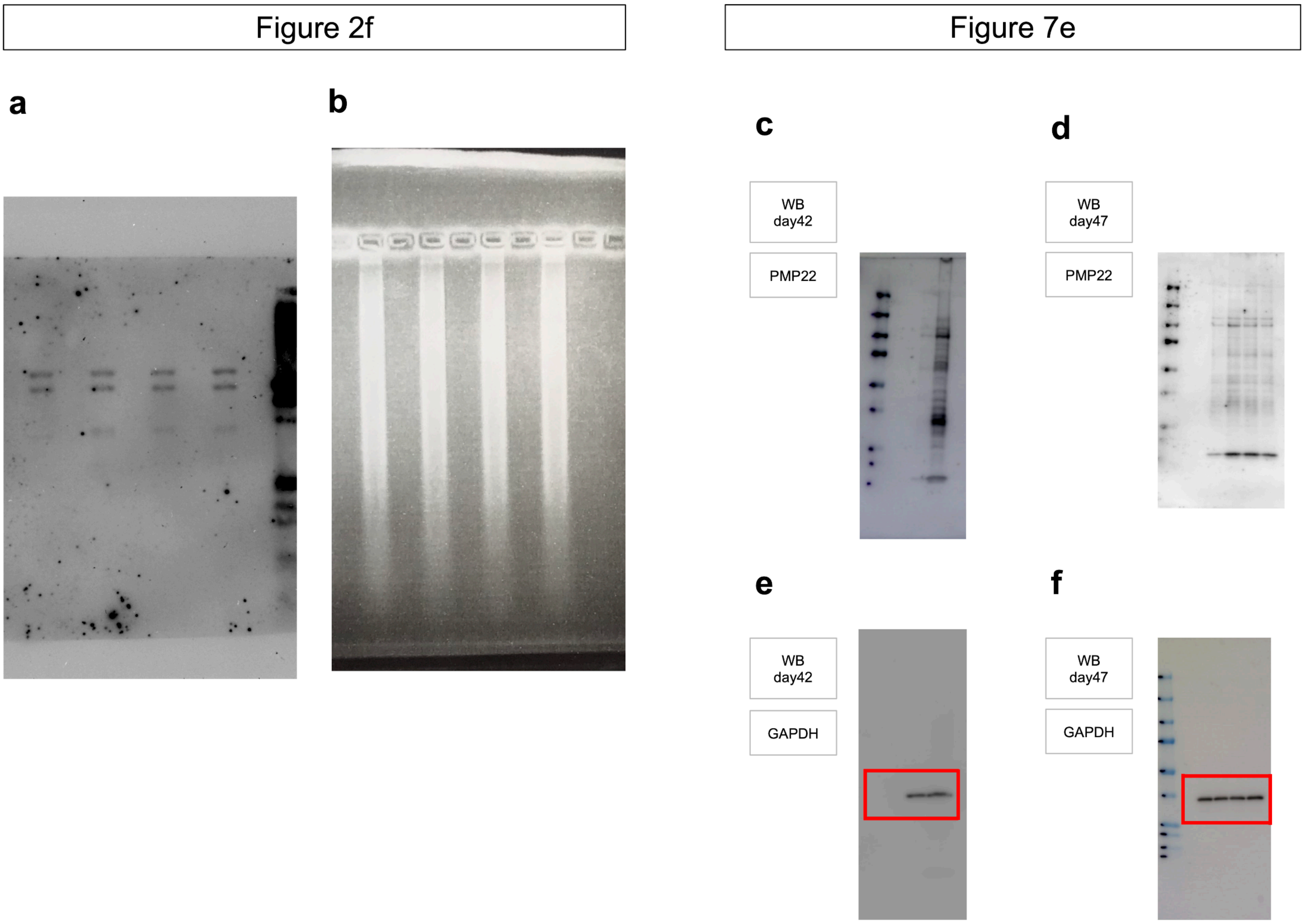

Supplementary Figure 9
Digital uncropped images of gel and blots
a) Southern blot corresponding to Figure 2f. b) Agar gel electrophoresis for Southern blot corresponding to Figure 2f. c) Immunoblot of PMP22 at Day 42 corresponding to Figure 7e. d) Immunoblot of PMP22 at Day 47 corresponding to Figure 7e. e) Immunoblot of GAPDH at Day 42 corresponding to Figure 7e. f) Immunoblot of GAPDH at Day 47 corresponding to Figure 7e.
