# Supplementary material for: AAV-mediated editing of PMP22 rescues Charcot-Marie-Tooth disease type 1A features in patient-derived iPS Schwann cells
Source: Commun Med (Lond). 2023 Nov 28;3:170. doi: 10.1038/s43856-023-00400-y (PMC10684506; doi:10.1038/s43856-023-00400-y)
Supplement: Supplementary file 4 — Description of Additional Supplementary Files [file 43856_2023_400_MOESM4_ESM.pdf]

Description of Additional Supplementary Files

File Name: Supplementary Data 1
Description: Source data for each experiment are shown in each page of the excel file as indicated with figure number
