# Supplementary material for: AAV-mediated editing of PMP22 rescues Charcot-Marie-Tooth disease type 1A features in patient-derived iPS Schwann cells
Source: Commun Med (Lond). 2023 Nov 28;3:170. doi: 10.1038/s43856-023-00400-y (PMC10684506; doi:10.1038/s43856-023-00400-y)
Supplement: Supplementary file 5 — Reporting Summary [file 43856_2023_400_MOESM5_ESM.pdf]

nature portfolio

Corresponding author(s): Hitoshi Okazawa

Last updated by author(s): Sep 30, 2023

Reporting Summary

Nature Portfolio wishes to improve the reproducibility of the work that we publish. This form provides structure for consistency and transparency in reporting. For further information on Nature Portfolio policies, see our Editorial Policies and the Editorial Policy Checklist.

Statistics

For all statistical analyses, confirm that the following items are present in the figure legend, table legend, main text, or Methods section.

n/a	Confirmed	
☐	☒	The exact sample size (*n*) for each experimental group/condition, given as a discrete number and unit of measurement
☐	☒	A statement on whether measurements were taken from distinct samples or whether the same sample was measured repeatedly
☐	☒	The statistical test(s) used AND whether they are one- or two-sided<br>*Only common tests should be described solely by name; describe more complex techniques in the Methods section.*
☐	☒	A description of all covariates tested
☐	☒	A description of any assumptions or corrections, such as tests of normality and adjustment for multiple comparisons
☐	☒	A full description of the statistical parameters including central tendency (e.g. means) or other basic estimates (e.g. regression coefficient) AND variation (e.g. standard deviation) or associated estimates of uncertainty (e.g. confidence intervals)
☐	☒	For null hypothesis testing, the test statistic (e.g. *F*, *t*, *r*) with confidence intervals, effect sizes, degrees of freedom and *P* value noted<br>*Give P values as exact values whenever suitable.*
☒	☐	For Bayesian analysis, information on the choice of priors and Markov chain Monte Carlo settings
☒	☐	For hierarchical and complex designs, identification of the appropriate level for tests and full reporting of outcomes
☒	☐	Estimates of effect sizes (e.g. Cohen's *d*, Pearson's *r*), indicating how they were calculated

Our web collection on statistics for biologists contains articles on many of the points above.

Software and code

Policy information about availability of computer code

Data collection	ImageJ ver.1.53 (NIH) was used for 2D image processing and acquisition of signal intensity.
Data analysis	GraphPad Prism 8 software (GraphPad Software, La Jolla, CA, USA) was used for statistical analyses and to plot data Python version 3.8 with Seaborn library was used to describe box-whisker plot.<br>R version 4.2.1 with exactRankTests library was employed to perform Wilcoxon's rank sum test using wilcox.exact function.

For manuscripts utilizing custom algorithms or software that are central to the research but not yet described in published literature, software must be made available to editors and reviewers. We strongly encourage code deposition in a community repository (e.g. GitHub). See the Nature Portfolio guidelines for submitting code & software for further information.

Data

Policy information about availability of data

All manuscripts must include a data availability statement. This statement should provide the following information, where applicable:

- Accession codes, unique identifiers, or web links for publicly available datasets
- A description of any restrictions on data availability
- For clinical datasets or third party data, please ensure that the statement adheres to our policy

We wrote the following sentences in "Data Availability" section.

March 2021

Human research participants

Policy information about studies involving human research participants and Sex and Gender in Research.

Reporting on sex and gender	Not applicable
Population characteristics	Not applicable
Recruitment	Not applicable
Ethics oversight	Not applicable

Note that full information on the approval of the study protocol must also be provided in the manuscript.

Field-specific reporting

Please select the one below that is the best fit for your research. If you are not sure, read the appropriate sections before making your selection.

☒ Life sciences ☐ Behavioural & social sciences ☐ Ecological, evolutionary & environmental sciences

For a reference copy of the document with all sections, see nature.com/documents/nr-reporting-summary-flat.pdf

Life sciences study design

All studies must disclose on these points even when the disclosure is negative.

Sample size	No sample size calculation was performed, and the sample size were similar to those reported in previous publications, PubMed ID 29397273, 27641503 and 34980925.
Data exclusions	There are no exclusion criteria for all analysis.
Replication	Experiments were independently repeated, the numbers of biological replicates are presented in the Figures.
Randomization	Simple randomization was performed to allocate samples and/or images to researchers before analysis.<br>The selection of images from immunohistochemistry/immunocytochemisty and the actual experiments of IHC/ICC were done by different researchers. In vitro live-cell imaging were done by different researchers.<br>Western blots are repeated until the necessary N was acquired.
Blinding	The information about group allocation or samples were opened to the data analyst or image acquisition researchers after finalizing results (m ake graphs etc).

Reporting for specific materials, systems and methods

We require information from authors about some types of materials, experimental systems and methods used in many studies. Here, indicate whether each material, system or method listed is relevant to your study. If you are not sure if a list item applies to your research, read the appropriate section before selecting a response.

Materials & experimental systems

n/a	Involved in the study	
☐	☒	Antibodies
☐	☒	Eukaryotic cell lines
☒	☐	Palaeontology and archaeology
☐	☒	Animals and other organisms
☒	☐	Clinical data
☒	☐	Dual use research of concern

Methods

n/a	Involved in the study	
☒	☐	ChIP-seq
☒	☐	Flow cytometry
☒	☐	MRI-based neuroimaging

Antibodies

Antibodies used	All antibodies used in the study are listed in the method.<br><br>The antibodies used for FACS were listed as following, MBP (M3821, Sigma Aldrich, St. Louis, MO, USA); mouse anti-Myelin Basic

	Protein antibody (ab62631, abeam, Cambridge, UK); Anti-Sl00 antibody (ab76749, abeam, Cambridge, UK).<br><br>The antibodies used for immunocytochemistry were dilution as following, rabbit anti-SlO0B antibody (1:500 ab52642, abeam, Cambridge, UK); goat anti-Soxl0 antibod y (1:100 sc-17342, Santa Cruz Biotechnology, Dallas, TX, USA); mouse anti-Myelin Basic Protein antibody (1:200 ab62631, abeam, Cambridge, UK); rabbit anti-MAP2 (1:100 sc-32791, Santa Cruz Biotechnology, Dallas, TX, USA); Alexa Fluor 488-conjugated anti-rabbit lgG (1:1000 A21206, Molecular Probes, Eugene, OR, USA); Alexa Fluor 568-conjugated anti-mouse lgG (1:1000 A10037, Molecular Probes, Eugene, OR, USA); Cy3-conjugat4ed anti-mouse lgG (1:500 705-165-003, Jackson Laboratory, Bar Harbor, ME, USA).<br><br>The antibodies used for electron microscopy were dilution as following, rabbit anti-CRISPR Cas9 antibody {1:25 ab203933, abeam, Cambridge, UK); mouse anti-Myelin Basic Protein antibody (1:50 ab62631, abeam, Cambridge, UK).
Validation	Activities of antibodies used for this study were validated via their manufacturer's Website.

Eukaryotic cell lines

Policy information about cell lines and Sex and Gender in Research

Cell line source(s)	HEK293 cells were purchased from TaKaRa (Kusatsu, Shiga, Japan)
Authentication	None of cell lines used were authenticated.
Mycoplasma contamination	All cell lines were negative for mycoplasma contamination.
Commonly misidentified lines (See ICLAC register)	We did not use any misidentified cell lines.

Animals and other research organisms

Policy information about studies involving animals; ARRIVE guidelines recommended for reporting animal research, and Sex and Gender in Research

Laboratory animals	The study did not involve any laboratory animals.
Wild animals	The study did not involve any wild animals.
Reporting on sex	Not applicable.
Field-collected samples	The study did not involve any samples collected from the field.
Ethics oversight	All animal experiments were performed in accordance with Animal Research: Reporting in vivo Experiments (ARRIVE) guidelines and were approved by the Committees on Gene Recombination Experiments and Animal Experiments of Tokyo Medical and Dental University (G2018-082C5 and A2023-113A).

Note that full information on the approval of the study protocol must also be provided in the manuscript.
